# Biomarkers in Diabetic Retinopathy and the Therapeutic Implications

**DOI:** 10.1155/2013/193604

**Published:** 2013-11-07

**Authors:** Katarzyna Zorena, Dorota Raczyńska, Krystyna Raczyńska

**Affiliations:** ^1^Department of Clinical and Experimental Endocrinology, Institute of Maritime and Tropical Medicine, Medical University of Gdańsk, Powstania Styczniowego 9b, 81-519 Gdynia, Poland; ^2^Department of Anesthesiology and Intensive Care Medicine, Medical University of Gdańsk, Poland; ^3^Department and Clinic of Ophthalmology, Medical University of Gdańsk, Poland

## Abstract

The main problem both in type 1 (T1DM) and type 2 (T2DM) diabetes is the development of chronic vascular complications encompassing micro- as well as macrocirculation. Chronic complications lower the quality of life, lead to disability, and are the cause of premature death in DM patients. One of the chronic vascular complications is a diabetic retinopathy (DR) which leads to a complete loss of sight in DM patients. Recent trials show that the primary cause of diabetic retinopathy is retinal neovascularization caused by disequilibrium between pro- and antiangiogenic factors. Gaining knowledge of the mechanisms of action of factors influencing retinal neovascularization as well as the search for new, effective treatment methods, especially in advanced stages of DR, puts special importance on research concentrating on the implementation of biological drugs in DR therapy. At present, it is antivascular endothelial growth factor and antitumor necrosis factor that gain particular significance.

## 1. Introduction

Dynamic increase in morbidity both for type 1 (T1DM) and type 2 (T2DM) diabetes has reached the state of an epidemic in developed countries and in the developing ones; the incidence of diabetes is increasing at still a quicker pace [1–3]. At present, 284.6 million people are sick with diabetes. According to statistics, in 2030, this number will increase to 438 million, that is, 6.4% of the global population [1]. The main problem both in type 1 (T1DM) and type 2 (T2DM) diabetes is the development of chronic vascular complications encompassing micro- as well as macrocirculation [4–7]. Chronic complications lower the quality of life and lead to disability. Moreover, they shorten life expectancy on average by 16 to 20 years in T1DM patients and by 4 to 6 years in those with type 2 diabetes [8–10]. It is also worth mentioning that the worldwide costs of treating diabetes and its complications are on average 5 to 10% of the overall funds for health service [11–13]. Diabetic retinopathy (DR) is the most common cause of vision loss, and a large number of diabetic patients experience significant vision impairment [10, 14, 15]. Within the first 10 years of living with diabetes, retinopathy can be diagnosed in nearly all T1DM patients and in over 60% of those with T2DM. In the *Wisconsin Epidemiologic Study of Diabetic Retinopathy *(WESDR), 3.6% of patients with T1DM diagnosed at a younger age and 1.6% of those in whom T2DM developed in a later stage of life were considered blind [15]. Moreover, in the group of T1DM patients, diabetic retinopathy was diagnosed as the cause of blindness in 86% of cases, while in T2DM group of patients, in whom the disease started at an older age and who often suffered from other diseases affecting sight, diabetic retinopathy caused loss of sight in 1/3 of cases [15].

Pathogenesis of DR is complex, and despite a lot of research, it has not been completely elucidated. The disease process encompasses characteristic changes, namely, thickening of the basement membrane coupled with its increased permeability, loss of pericytes leading to diminished vessel wall tone and development of protruding micro aneurysms, and proliferation of mesangium causing obstruction and obliteration of capillaries. Now, we know that diabetic retinopathy is the effect of genetic, environmental, and immunological factors acting together [16–18]. The main initiating factor for the changes observed in the course of diabetic retinopathy is hyperglycemia. Its metabolic consequences stem from toxic effects of chronically heightened glucose levels in the blood. Numerous trials have demonstrated that chronic, nonphysiologically high blood glucose concentration in diabetic patients causes pericyte damage in the retina, as these cells are the ones most quickly reacting to the glucose overflow. The damaging effect within parietal cells is done by the activation of several metabolic pathways. One of the most important biochemical reactions, which plays an important part in the development of vascular complications in the course of diabetes, is the nonenzymatic glycation of proteins. Its effects are advanced glycation end products (AGEs) [19, 20]. AGEs are permanent, non-reversible products with an ability to produce cross-links between proteins [19–21]. Animal experiments in vitro and in vivo have proven the relationship between AGE concentration and the presence of symptoms characteristic for diabetic retinopathy. The researchers have put particular attention to the enhancement of apoptosis of cells building retinal vessels walls, the formation of cellular capillaries in the retina, and the retinal neovascularization [22, 23]. It is worth noting that the application of AGE inhibitors prevented abnormal pathogenesis [24, 25]. Despite many trials, the mechanisms of enhanced retinal pericytes apoptosis remain unclear. However, numerous pieces of research suggest that such enhanced apoptosis stems from the accumulation of AGE in pericytes. AGEs exert toxic proprieties and may change cellular enzymes' activity. AGEs accumulating in the basal membranes of the retina modify vascular basal membranes by creating cross-links. As a consequence, pathological changes develop in the retinal vessels [16, 26–28]. 

**Figure 1:**
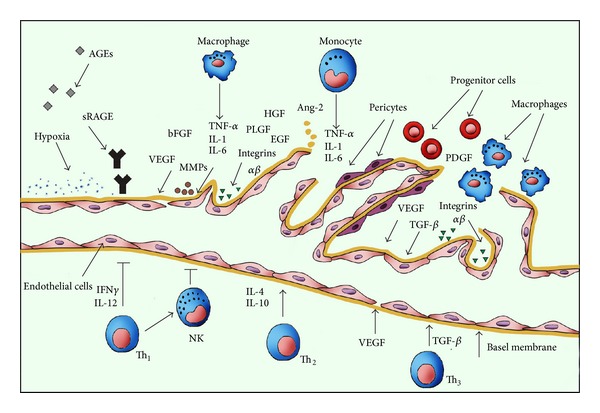
Biomarkers involved in the development and progression of diabetic retinopathy. AGEs—advanced glycation end products, RAGEs—receptor for advanced glycation end products, bFGF—basic fibroblast growth factor, VEGF—vascular endothelial growth factor, MMPs—metalloproteinases, TNF-*α*—tumor necrosis factor alpha, IL1—Interleukin 1, Interleukin 6—IL6, HGF—hepatocyte growth factor, PLGF—placental growth factor, EGF—epidermal growth factor, Ang-2—angiopoietin-2, PDGF—platelet-derived growth factor, TGF-*β*—transforming growth factor-beta, Th—helper lymphocytes, NK—natural killer, IL4—Interleukin 4, IL10—Interleukin 10, IL12—Interleukin 12, IFN*γ*—interferon-*γ*. Modified Figure 1 of [16].

Up till now, both our research and the work of other authors show significantly higher AGEs level both in the serum of children with T1DM and late vascular complications [29, 30] and in adults patients with diabetes and PDR [31, 32]. Moreover, in our last research, by applying ROC curve for AGEs, we have determined the reference level for 19.867 pg/mL in the examined group of children and adolescents with T1DM [29]. The end products of advanced glycation influence the cells of many tissues by specific receptors localized on macrophages and endothelial cells which take part in the metabolic turnover of proteins, tissue remodeling, and inflammatory process [23, 24]. The best known receptor linking the advanced glycation end products is the receptor for advanced glycosylation end products (sRAGE). AGE interaction with sRAGE on the surface of monocytes, macrophages, and endothelial cells increases the synthesis and secretion of pro-inflammatory cytokines, such as interleukin 1 (IL1), tumor necrosis factor *α* (TNF-*α*) and vascular endothelial growth factor (VEGF), adhesive molecules, and the activation of a nuclear transcription factor NF*κ*B [33–36]. This process encompasses characteristic changes, namely, thickening of the basement membrane coupled with its increased permeability, loss of pericytes leading to diminished vessel wall tone, and development of protruding micro aneurysms, as well as proliferation of mesangium causing obstruction and obliteration of capillaries [16, 21, 23], Figure 1.

Research on the drug which would protect amino groups of proteins exposed to glycation in the first step or prevent the cross-links formation in the third step of the reaction is still ongoing. Drugs preventing the formation of AGE complexes probably block the carbonyl groups of Amadori rearrangement products. This prevents the formation of cross-links with other proteins by binding with their amino groups. Aspirin also exhibits some protecting properties [37, 38]. Another anti-AGE agent, pyridoxamine, also prevented development of DR. However, clinical trials of anti-AGE agents for the treatment of DR have not yet been conducted. Based on those reports, we evaluated the effects of oral aminoguanidine and pyridoxamine on the development of cataract and DR in SDT rats. Authors reported that aminoguanidine prevented accumulation of CML and resulted in almost complete inhibition of DR [39, 40].

## 2. Vascular Endothelial Growth Factor (VEGF) 

VEGF, also known as the vascular permeability factor—VPF or vasculotropin, is nowadays considered the main angiogenesis-controlling factor. VEGF is produced by endothelial cells, macrophages, CD4 lymphocytes, plasma cells, myocytes, megakaryocytes, and neoplastic cells [41]. It is a 45 kDa homodimeric glycoprotein belonging to a wide family of growth factors. At present, the VEGF family consists of 6 proteins: VEGF-A,-B,-C,-D,-E, and placental growth factor (PGF) [41]. The best known and most widely used in clinical practice is VEGF-A. We also know several VEGF isoforms: VEGF121, VEGF145, VEGF148, VEGF162, VEGF165, VEGF183, VEGF189, and VEGF206, differing by the length of aminoacids chain, the ability to bind with heparin, mitogenic activity, and the affinity to VEGF receptors [42, 43]. The role of VEGF in the pathogenesis of diabetic retinopathy was first confirmed in 1994 [44]. VEGF stimulates proliferation and migration of endothelial cells and increases vascular permeability. Besides, it induces the production of tissue collagenase and increases macrophage and monocyte chemotaxis. It is also said that VEGF contributes to the increased permeability of blood-retina barrier and that it stimulates the neovascularization process in the advanced retinopathy [43, 45–47]. The increased level of VEGF expression was found already in the early stages of nonproliferative retinopathy in children and adolescents with T1DM [48–50]. It is been suggested that VEGF may play a part in the development of vascular changes in children and adolescents already in the first years of diabetes, when popular and available diagnostic methods would not yet show any changes characteristic of diabetic retinopathy [49, 50]. Moreover, in our other research (Zorena et al. 2010) we have shown that the level of VEGF was higher in patients with T1DM diagnosed with retinopathy, nephropathy, and hypertension as compared with patients with T1DM, retinopathy, and nephropathy but with no hypertension. What is more is that there were also no significant differences in the serum levels of VEGF between the group of patients with T1DM, retinopathy, and nephropathy but no hypertension and the healthy control level. These data show that until the appearance of three complications, VEGF level in children and adolescents with T1DM is not significantly higher as compared with healthy controls [51]. Furthermore, there are numerous works in literature showing increased values of VEGF both in the vitreous humor and in the vitreous body of patients with proliferative diabetic retinopathy or diabetic macular edema (DME) as compared with healthy controls [45–47].

## 3. Antivascular Endothelial Growth Factor (VEGF) Treatment

Currently, there are four anti-VEGF agents which have been used in the management of diabetic retinopathy, including pegaptanib (Macugen; Pfizer, Inc., New York, USA), ranibizumab (Lucentis; Genentech, Inc., South San Francisco, California, USA), bevacizumab (Avastin; Genentech, Inc.), and VEGF Trap-Eye (Regeneron Pharmaceuticals, Inc., Tarrytown, New York, USA).

### 3.1. Pegaptanib

(Macugen; Eyetech Pharmaceuticals, Inc. and Pfizer Inc., New York) is an aptamer, which was the first of anti-VEGF factors to be approved for the treatment of neovascular AMD, as an inhibitor of the 165 VEGF isomer [52]. A phase 2/3, randomized, double-blind, and 2-year trial has been performed to assess the safety and efficacy of intravitreal pegaptanib sodium 0.3 mg compared with sham injections in subjects with DME, with focal/grid photocoagulation being permitted as needed after week 18. The authors showed that intravitreal pegaptanib sodium 0.3 mg was well tolerated and demonstrated superior efficacy over the sham treatment in the therapy of patients with DME. The proportion of patients with ≥10 letters (or 2 lines) of visual acuity improvement at week 54 was statistically significantly greater in the pegaptanib group versus those in the sham treatment arm [53]. 

### 3.2. Bevacizumab

 (Avastin; Genentech, South San Francisco, California) is 93% of a human immunoglobulin Ig1 and 7% of a murine fragment. A specific antibody domain recognises all VEGF-A isoforms [54, 55]. Bevacizumab has recently been used by ophthalmologists in an offlabel use as an intravitreal agent in the treatment of proliferative eye diseases, particularly for choroidal neovascular membrane (CNV) in AMD. Although not currently approved by the FDA for such use, the injection of 0.75 mg–2.5 mg of bevacizumab into the vitreous cavity has been performed without significant intraocular toxicity [56]. Many retina specialists have noted impressive results in the setting of CNV, proliferative diabetic retinopathy, neovascular glaucoma, diabetic macular edema, retinopathy of prematurity, and macular edema secondary to retinal vein occlusions [57, 58]. What is interesting is that in the last work of Suzuki et al. 2013, after bevacizumab injection three days before vitrectomy, apart from VEGF reduction, the authors also showed lower levels of IL-1RA, IL-5, IL-10, IL-12, IL-13 cytokines, and IFN-*γ* [59]. Research of DRCR.net group continues to analyze the possibility of using bevacizumab in the treatment of diabetic retinopathy [60, 61].

### 3.3. Ranibizumab

(Lucentis; Genentech, South San Francisco, California) is a humanized antibody fragment directed at all isoforms of VEGF-A and is fabricated specifically for intravitreal use. Ranibizumab is now FDA approved for the treatment of age-related macular degeneration as well as macular edema associated with retinal vein occlusion. For diabetic macular edema, an initial small pilot study showed efficacy of intravitreal injections of ranibizumab in reducing macular thickness and improving visual acuity [62, 63]. 

In a recent study, authors presented a two-year observation of patients after dosing ranibizumab in diabetic macular edema. After the initial 6 months, all patients were followed up every 2 months. Patients in group 1 could be reinjected if they had persistent or recurrent DME, patients in group 2 could receive either ranibizumab alone or laser only, and patients in group 3 could receive ranibizumab alone or in combination with laser. After 24 months, patients gained 7.7, 5.1, and 6.8 letters in each of the groups, respectively, and the percentage of patients who gained three or more lines of visual acuity was 24, 18, and 26%, respectively [64]. A recent study presented Brown et al. 2013 to report 36-month outcomes of RIDE (NCT00473382) and RISE (NCT00473330), trials of ranibizumab in diabetic macular edema [65]. Patients were randomized equally (1 eye per patient) to monthly 0.5 mg or 0.3 mg ranibizumab or sham injection. In the third year, they were eligible to cross over to monthly 0.5 mg ranibizumab. The strong visual acuity (VA) gains and improvement in retinal anatomy achieved with ranibizumab at month 24 were sustained through month 36. Ocular and systemic safety were generally consistent with the results seen at month 24 [65].

## 4. Vascular Endothelial Growth Factor Trap-Eye

VEGF Trap is a 115 kDa recombinant fusion protein consisting of the VEGF binding domains of human VEGF receptors 1 and 2 fused to the Fc domain of human IgG1 [66]. The research on the VEGF Trap is now approaching the end of phase II in the treatment of retinal neovascularisation secondary to AMD. Moreover, phase II of the research on using this substance in the treatment of a diabetic eye disease (DED) also starts. A phase I study showed that a single intravitreal injection of VEGF trap-eye exerted biological activity by improving visual acuity and reducing excess retinal thickness in eyes with DME [63]. In this phase II randomized clinical trial, intravitreal VEGF trap-eye was superior to macular laser treatment by the modified ETDRS protocol for the treatment of DME over a 24-week period. VEGF trap-eye resulted in significantly better mean visual acuity outcomes (+8.5 to +11.4 versus +2.5 letters gained) and greater mean reductions in retinal thickness (−127.3 to −194.5 *μ*m versus −67.9 *μ*m) compared with laser alone [67].

It is worth noting that all the above mentioned drugs have their side effects depending on the drug and the method of administration itself [68, 69].

## 5. Insulin-Like Growth Factor (IGF)

IGF is a polypeptide showing likeness to insulin. There are two such factors: insulin-like growth factor I (IGF-I) and insulin-like growth factor II (IGF-II). IGF-1 polypeptide circulates in the blood as an IGF-binding protein (IGF-BP), probably inhibiting the activity of the free IGF. IGF-I is the main growth factor secreted under the influence of a human growth hormone hGH. In vivo and in vitro studies indicate that IGF-1 acts as an antiapoptotic and anti-inflammatory factor [70, 71]. In ischemic rat kidneys, IGF-I has been shown to exert a protective activity by inhibiting pro-inflammatory cytokines [72], while in Parkinson's disease, antiapoptotic IGF-I acted by inhibiting a GSK-3 beta signaling pathway [71]. Protecting IGF-1 action encompasses also central nervous system and cardiac myocytes. Postnatal IGF-1 deficit may also play a part in the development and worsening of neurological deficits in preterm babies [73]. Moreover, in neonates born before term, low IGF-1 concentration is a causative factor for the development of retinopathy of prematurity (ROP) [74]. On the other hand lower IGF-1 levels have been observed in children and adolescents with T1DM and microangiopathy as compared with those with T1DM but no microangiopathy [75, 76]. Moreover, IGF-I concentration was lowest in those children and adolescents with T1DM who had been suffering from diabetes for more than ten years. Interestingly, the same children had higher level of VEGF in serum, and that level had been rising in parallel with the duration of the disease, being highest in patients living with the disease for more than 10 years [48, 75, 77]. In adult patients with PDR, the levels of both IGF-1 and VEGF in the vitreous body were higher than in the control group [78, 79]. It is worth noting that the authors of these studies did not observe differences in IGF-1 or VEGF levels in the serum. On one hand, this effect can be explained by the increase of the IGFBP's concentrations in the vitreous body, which in turn neutralizes the increased IGF-1 production, and on the other hand, it can be explanied by inhibiting the production of free IGF-1 in the tissues of diabetic patients [80].

## 6. Pigment Epithelium Derived Factor (PEDF)

PEDF is a 50 kDa protein with neuroprotective, neurotrophic, and antiangiogenic activities [81, 82]. Pigment epithelium-derived factor is a glycoprotein that belongs to the superfamily of serine protease inhibitors. It was first purified from a conditioned media of human retinal pigment epithelial cells with neuronal differentiating activity [83]. Recently, PEDF has been shown to be the most potent inhibitor of angiogenesis in the mammalian eye; it inhibited retinal endothelial cell growth and migration and suppressed ischemia-induced retinal neovascularization [83–85]. In studies on mice without PEDF, gene Doll et al. have shown that lack of this gene results in serious disturbances both in cell differentiation and in retina architecture [84]. The hypothesis that PEDF may inhibit angiogenesis by directly diminishing the expression of the vascular endothelial growth factor gene first appeared in 2003 [86]. PEDF also inhibits the production of reactive oxygen species (ROS) and the monocyte chemotactic protein (MCP-1). It also neutralizes harmful effects of the glycation end products. Newest data confirm that PEDF exhibits also a direct effect on vascular endothelial growth factor receptor 1 (VEGFR-1) by increasing the gamma-secretase complex activity [87]. Other research suggests the role of a transcription factor NF-*κ*b and a Fas ligand, a cytokine belonging to the TNF and its receptor (FasFas/CD95) superfamily, by PEDF action. Volpert et al. have proven that anti-FasL antibodies and the use of caspase inhibitors inhibit PEDF. So, PEDF can prevent cell apoptosis by activating transcription factor NF-*κ*b, as well as activate programmed cell death by increasing the expression of a Fas ligand [88]. In experimental studies, it is been shown that PEDF inhibits neoangiogenesis when oxygen concentration in the blood is normal but promotes it when oxygen is scarce [89]. In the vitreous body of diabetic patients with PDR, the level of a soluble vascular endothelial growth factor receptor-1 (sVEGF-R1) has been significantly higher, and the level of PEDF was lower as compared to the control group of patients with diabetes but no signs of retinopathy [90]. 

PEDF may exhibit antiangiogenic effect through its antioxidant action. The authors have shown that by its antioxidant effects PEDF can block the effects of proangiogenic factors [91, 92]. In animal models, it is been shown that the administration of PEDF may alleviate characteristic changes in diabetic retinopathy [91]. In recent studies, Ishibashi et al. (2013) demonstrated for the first time that PEDF could block the AGE-induced apoptotic cell death of podocytes by suppressing RAGE expression and subsequent ROS generation partly via PPAR*γ* activation [92]. 

## 7. Transforming Growth Factor Beta (TGF*β*)

TGF-*β* belongs to the family of transforming growth factors with immunoregulatory properties [93]. In humans, transforming growth factor *β* (TGF-*β*) is present in three inactive isoforms bound with latent associated protein (LAP) and latent TGF-beta binding protein (LTBP). TGF-*β*1 is present in endothelial cells, hemopoietic cells, and connective tissue cells, TGF-*β*2 in epithelial tissue and in neurons, and TGF-*β*3 in connective tissue cells. The activation takes place with the use of plasmin or cathepsin D, after cleavage from nonactive complex. It exerts its action through type I, II, and III TGF-*β* receptors and in conjunction with a specific SMAD protein present in the cytoplasm [94–96]. Its biological role consists of stimulating mesenchymal cell division which in turn enhances angiogenesis and chondrogenesis. TGF-*β* inhibits proliferation of T and B lymphocytes, NK cells and the expression of class II MHC particles as well as the formation of cytotoxic T-lymphocytes [97]. Moreover, it stimulates, for example, posttraumatic regenerative processes by increasing the production of proteins, collagen, fibronectin, and integrin in fibroblasts. TGF-*β* can inhibit enzymes that take part in the degradation of these proteins—heparinase, collagenase, and stromelysin. Moreover it increases the production of tissue inhibitors of metalloproteinases (TIMP). The increased level of TGF-*β*1 has been found in the idiopathic pulmonary fibrosis, diabetic retinopathy, and glaucoma [97–99]. Among the drugs currently in clinical use, the few that have anti-TGF-activity include tranilast, losartan, glitazones, and imatinib mesylate [100, 101]. These drugs have been found to block the production, activation, or biological activity of TGF-*β*. Moreover, in the presence of trans-resveratrol showed inhibition of TGF-*β*1 and VEGF, COX-2, IL-6 and IL-8 [101]. However, the total blockade of TGF-*β* function can have adverse effects, such as the exposure of intraocular tissue to the damaging effects of local and systemic immune responses. Therefore, a new anti-TGF-*β* therapy is to selectively block its activation at sites where excess TGF-activation occurs, without affecting its basic function [100].

## 8. Interleukin 12 (IL12)

IL-12 is a multipurpose cytokine and in physiological conditions, it is produced mainly by macrophages, dendritic cells, keratinocytes, granulocytes, and mast cells [102]. It stimulates proliferation, activation, and cytotoxicity of lymphocytes T and NK (*natural killer*) cells, as well as the production of INF*γ* and TNF-*α* by these cells. On one hand, IL-12 contributes to the development of autoimmunological diseases, such as rheumatoid arthritis, multiple sclerosis, and type 1 diabetes [103, 104]; on the other, in vitro and in vivo studies have shown that this cytokine has strong antineoplastic activities [105]. Few studies conducted recently indicate that IL-12 may have antiangiogenic properties [106]. In vitro studies have shown that maintaining the equilibrium between pro- and anti-inflammatory mediators allows for maintaining physiological angiogenesis, while disturbing this equilibrium in favor of the first leads to pathological angiogenesis [107, 108]. In our studies (Zorena et al.), we have shown that in the group of children with T1DM and retinopathy the serum level of TNF-*α* was significantly higher and the level of IL-12 was significantly lower than in the control group without the symptoms of diabetic retinopathy [108]. Obtained results suggest that increased TNF-*α* production may be the result of insufficient IL-12 level. Maybe the balance between the pro- and antiangiogenic cytokines is one of the factors preventing the development of diabetic nephropathy and retinopathy in those children. It has been shown that, in T1DM patients, IL-12 serum concentration was the highest in subgroup with > or = 3 mg/L hsCRP (*P* < 0.05) [109]. Moreover, a significantly higher concentration of proinflammatory cytokine IL-12 has been found in the aqueous humor of nontreated diabetic retinopathy patients in comparison with diabetic patients treated for retinopathy, without retinopathy, or with healthy individuals [110]. The attempts of therapy with rhIL-12 antibodies (ustekinumab) have been performed in patients with Crohn's disease and psoriatic arthritis [111, 112].

## 9. Tumor Necrosis Factor-Alpha (TNF-*α*)

TNF-*α* is one of the main inflammatory response cytokines. It is produced mainly by monocytes and macrophages and interacts with them by endo-, para-, and autocrine mode of action. It acts chemotactically on monocytes and neutrophils and activates them as macrophages. It enhances the cytotoxicity of monocytes and macrophages, being at the same time one of the mediators of this cytotoxicity. Biological effects largely depend on the quantity and intensity of TNF secretion [113]. TNF-*α* is one of the cytokines inducing the interruption of the blood-retina barrier by loosening tight junctions between individual endothelial cells of the retina and also between the cells of the retinal pigment epithelium. Apart from taking part in the inflammatory processes, TNF-*α* plays an important role in neovascularisation and vasomotor reactions [114, 115]. Factors significantly enhancing the secretion of the cytokine are hypoxia and methylglyoxal-modified proteins, which increase the level of mRNA TNF-*α* expression. This cytokine fulfills its numerous duties thanks to the ability to stimulate the synthesis of other cytokines, functionally connected with TNF-*α*, extracellular matrix proteins, modulation of monocyte, and macrophage chemotaxis, as well as the effect on the expression of adhesion molecules in the retinal vessels [114]. TNF-*α* has been found in the serum of children and adolescents with T1DM and NPDR [49, 116]. The same authors have shown that out of the examined proinflammatory factors, serum TNF-*α* level can be an independent predictive factor for NPDR development in children with T1DM [116]. Similarly higher level of serum TNF-*α* have been found in adult patients with T1DM and PDR [117, 118]. Increased levels of TNF-*α* has also been found in the vitreous body of patients with T2DM and PDR as compared with the control group [119].

Currently, five anti-TNF agents are commercially available: etanercept, infliximab, adalimumab, ceertolizumab pegol, and golimumab [120–136].

### 9.1. Etanercept (Enbrel, Pfizer, New York, NY, USA)

It is a fusion (hybrid?) protein composed of a TNF receptor and the Fc fragment of human IgG antibody. It inhibits the binding of TNF-*α* and TNF-*β* to the surface TNF receptors, inactivating TNF and suppressing neutrophil migration and proinflammatory cytokine synthesis. Clinical studies have been indeterminate regarding the efficacy of etanercept for the treatment of ocular inflammation [120–122].

### 9.2. Infliximab (Remicade, Janssen, Beerse, Belgium)

It is an IgG1 chimeric monoclonal antibody with a constant human region and a variable murine one. This agent binds both the soluble and the cell-bound TNF-*α* but not TNF-*β* [123]. It has shown encouraging responses in patients with treatment-resistant ocular inflammation including Behçet's disease, Wegener's granulomatosis, sarcoidosis, and juvenile inflammatory arthritis [124]. However, recently, the illustrated paper demonstrates a rare extraintestinal manifestation of Crohn's disease, orbital myositis, and its temporal relationship to the discontinuance of infliximab therapy and its successful treatment, without recurrence with tapering prednisone and adalimumab [125].

### 9.3. Adalimumab (Humira, Abbott)

It is a fully humanized IgG1 monoclonal antibody, specifically directed against TNF-*α*, which binds both its soluble and cell-bound forms [126]. Adalimumab has been used with increasing frequency and found to be effective for treatment of birdshot retinochoroidopathy, juvenile inflammatory arthritis, Behçet's disease, and diabetic macular edema [127, 128]. 

### 9.4. Rituximab (Rituxan, Biogen Idec, Weston, MA)

It is a chimeric monoclonal antibody that binds to CD20 antigen on the surface of B cells and suppresses B-cell differentiation resulting in reduced IgG and IgM production [129]. It has been found to be effective in treatment of systemic lupus erythematosus, Behçet's disease, Wegener's granulomatosis uveitis, and retinal vasculitis [130, 131]. 

### 9.5. SIMPONI (Golimumab, Janssen Biotech, Inc.)

It is a human monoclonal antibody forming stable complexes with high affinity to the soluble and transmembrane form of human tumor necrosis factor (TNF-*α*), preventing TNF-*α* binding with its receptors. Human TNF binding by golimumab neutralizes TNF-*α*-induced expression of adhesive selectin E particles, vascular cell adhesion molecules (VCAM-1), and intercellular adhesion molecules (ICAM-1) on the surface of endothelial cells. In in vitro studies, golimumab inhibits the TNF-induced secretion of interleukin IL-6, IL-8 and the granulocyte-macrophage colony stimulating factor (GM-CSF). In patients receiving golimumab, there was an improvement in C-reactive protein concentration, which in turn significantly decreased concentrations of interleukin 6 (IL-6), ICAM-1 particles, metalloproteinase (MMP-3), and vascular endothelial growth factor (VEGF). Moreover, in patients with RA or ankylosing spondylitis, it was the TNF-*α* concentration that decreased, while in those with psoriatic arthritis, it was the IL-8 concentration that decreased [132–134]. However, it should be noted that the above-mentioned administration of monoclonal antibodies may bind to ocular complications [135, 136].

The search for new, efficient treatment methods, especially in advanced stages of diabetic retinopathy, makes the research on biological drugs especially important. Conducted studies suggest that among known factors, both pro-angiogenic (such as TNF-*α* or VEGF) and antiangiogenic, PEDF is a good material for study; although at present, the success of monoclonal antibody therapy can be judged to be merely moderate. The administration of all the above mentioned drugs is associated with many complications, drugdependent as well as linked with the method of drug administration itself. The latter can give transient complications, such as subconjunctival hemorrhage, the feeling of a foreign body under the eyelid, and increased intraocular pressure. Serious complications, with about 0.05% frequency, encompass the intraocular inflammation, retinal detachment, damage to the lens, or hemorrhage into the vitreous body. However, ongoing energetic studies in many centers around the world suggest that most probably in the near future we will achieve therapeutic success in preventing loss of sight in DM patients.
